# Tobacco, biological aging and epigenetic resilience: the Andorran paradox

**DOI:** 10.3389/fragi.2026.1839065

**Published:** 2026-05-26

**Authors:** Pooja Reddy, Dhairya Nanavaty, Pradeepkumar Devarakonda, Pedro Moreno

**Affiliations:** 1 Georgetown University, Washington, WA, United States; 2 Mount Sinai Hospital, New York, NY, United States; 3 University of Missouri, Columbia, DC, United States

**Keywords:** Andorra, DNA methylation, epigenetic resilience, healthy aging, longevity paradox, tobacco

## Abstract

Longevity is not solely determined by the absence of risk factors but often reflects the balance between harmful exposures and protective adaptations. We introduce the framework of epigenetic resilience as a counterbalancing mechanism by which favorable environmental and lifestyle factors can potentially offset detrimental influences such as tobacco use. Tobacco is a well-established accelerator of biological aging through adverse epigenetic reprogramming, yet populations such as Andorra paradoxically exhibit both high smoking prevalence and some of the longest life expectancies worldwide. This observation underscores the potential role of resilience factors—including diet, physical activity, psychosocial cohesion, and environmental context—that may buffer or counteract the negative epigenetic imprints of smoking. By highlighting this paradox, we emphasize the importance of understanding how protective influences reshape DNA methylation landscapes to sustain healthspan despite harmful exposures. Recognizing and enhancing epigenetic resilience might offer a path toward targeted interventions that could mitigate the consequences of tobacco and other stressors, ultimately informing strategies to promote healthy aging at both individual and population levels.

## 1 Introduction

### 1.1 The Andorran way of living, the importance of tobacco, and the Andorran paradox

#### 1.1.1 The Andorran way of living

Nestled in the heart of the Pyrenees, Andorra enchants with its breathtaking mountain vistas and crystal-clear valleys. Its blend of natural beauty and old-world charm makes it a hidden gem between Spain and France.

Life in Andorra moves at a peaceful, unhurried pace, surrounded by nature’s serenity and fresh alpine air. Andorrans maintain a strong sense of community and family cohesion, reflected by a low divorce rate of 2.7% in 2023 World Population Review (2025). Despite its reliance on buses for public transport, Andorra’s small size and extensive walking and hiking trails make active mobility common. Popular recreational activities include road cycling, mountain biking, and skiing, supported by an emphasis on physical activity within schools. The country is characterized by high socioeconomic status, low taxes, minimal unemployment, the absence of homelessness, and excellent healthcare access. Andorrans take pride in their Catalan heritage, with Catalan as the official language, while Spanish and French are also widely spoken (Everyculture, 2025).

Blue Zones, first described by Poulain et al. (2004) in Experimental Gerontology, represent geographic regions with exceptionally high concentrations of centenarians and prolonged healthspan (Poulain et al., 2004). These regions share nine evidence-based lifestyle factors, collectively termed the “Power 9,” associated with enhanced longevity (Buettner and Skemp, 2016). Although Andorra is not formally classified as a Blue Zone, its national ethos embodies many of these principles.

### 1.2 Importance of tobacco in Andorra

Tobacco has been central to Andorra’s history and economy since the 17th century, when cultivation flourished through cross-border smuggling into Spain and France. The microstate’s unique tax and regulatory status allowed tobacco farming and trade to thrive well into the 20th century. For decades, tobacco represented the most important industry in Andorra, until tourism overtook it. It remains a livelihood for farmers and a cultural symbol, exemplified by the Reig family’s tobacco factory, which was converted into a museum and is now one of the most popular tourist destinations (d’Argemir, 2025; Museum, 2021; Population, 2025; World Population Review, 2025; World Population, 2025).

Despite tourism’s rise, tobacco retains economic and cultural significance. Andorra ranks among the top ten countries globally for tobacco consumption World Population, 2025. According to Andorran statistics from 2022, 33.3% of men and 33.8% of women smoke daily. Trends show that prevalence among men has decreased from 43% in 2000, while prevalence among women has risen from 28% in 2000. In 2021, tobacco was responsible for 25.2% of all deaths in Andorra (Andorra, 2025).

### 1.3 The Andorran paradox

This heavy consumption stands in paradoxical contrast to health outcomes. United Nations (UN) data estimate Andorran life expectancy at 84 years (82 for men, 86 for women), ranking fifth highest worldwide (c), while a 2015 Lancet analysis of Sustainable Development Goals placed Andorra fourth globally for overall health outcomes (Lim et al., 2016). Compared to other high-income nations with similar GDP *per capita* and lower tobacco prevalence, Andorra’s longevity is striking. This tension between high-risk exposure and exceptional survival forms the foundation of the Andorran paradox.

### 1.4 Epigenetic resilience

We introduce epigenetic resilience to contextualize the Andorran paradox, emphasizing that biological aging reflects the balance between adverse exposures and stabilizing environmental conditions. This framework positions longevity as an emergent property of epigenetic stability maintained despite risk.

## 2 Methodology

This perspective was developed through a targeted narrative review of the published literature and publicly available population-level data. A structured literature search was conducted in PubMed and Google Scholar to identify studies related to biological aging, epigenetic clocks, DNA methylation markers associated with smoking, and lifestyle or socioeconomic factors influencing epigenetic aging. Priority was given to original research articles, systematic reviews, and large population-based cohort studies where available.

Population-level data on life expectancy, smoking prevalence, healthcare access, and socioeconomic indicators for Andorra were obtained from publicly available international databases and reports from global health and development organizations, including the United Nations, Global Burden of Disease studies, and World Population Review. These sources were used descriptively to contextualize national-level trends and were not subjected to statistical analysis.

No new experimental data were generated, and no individual-level analyses were performed. The manuscript presents a hypothesis-generating conceptual framework based on the synthesis of existing evidence rather than causal inference.

Accordingly, the interpretations presented should be viewed as exploratory and intended to guide future hypothesis-driven and mechanistic studies.

## 3 Biological aging and the role of epigenetic clocks

### 3.1 Understanding biological aging

Chronological age refers to the number of years a person has lived (Yaneske and Angione, 2018), whereas biological aging reflects the cumulative molecular and cellular damage accrued over time (Bel et al., 2022). Biological aging encompasses genetic, environmental, and metabolic aspects of an individual’s lifespan and hence is preferentially used over chronological aging (Mathur et al., 2024).

The aging process is derived from the complex interaction of genetic, epigenetic, and environmental factors. Lopez-Otin et al. described “Hallmarks of Aging,” which included epigenetic alterations, telomere attrition, genomic instability, mitochondrial dysfunction, cellular senescence, decline in proteostasis, impaired macro-autophagy, stem cell exhaustion, altered intracellular communication, chronic inflammation, nutrient sensing dysregulation, and dysbiosis (López-Otín et al., 2023).

Cellular senescence, a hallmark of aging, involves irreversible cell cycle arrest and the acquisition of a senescence-associated secretory phenotype (SASP). This process contributes to tissue dysfunction, chronic inflammation, and age-related morbidity. It is implicated in the pathogenesis of various cardiovascular diseases, including atherosclerosis (stroke, peripheral arterial disease, and coronary artery disease), valvular heart diseases, arrhythmias, and hypertension. Drugs halting this process, known as senolytics, are being studied in preclinical trials as a potential treatment option for cardiovascular diseases (Kumar et al., 2024).

Similar to cellular senescence, all the other individual hallmarks of aging have been implicated in the pathogenesis and worsening of multiple chronic conditions related to cardiovascular diseases, cancers, etc. Diagnostic tools and therapeutic tools are under study to potentially aid in the treatment of these diseases by modifying and possibly reversing biological aging (Fuster, 2024).

### 3.2 Measuring biological aging through epigenetic clocks

Biomarkers, or “aging clocks,” have been developed as biological age predictors. Among these, epigenetic clocks are one of the earliest, most widely used biomarkers with the most robust data. They measure changes in DNA methylation patterns—specifically age-related methylation of cytosine–phosphate–guanine (CpG) sites across the genome (Horvath, 2013; Hannum et al., 2013). To put in context, CpG sites are regions of DNA where a cytosine nucleotide is followed by a guanine nucleotide, and DNA methylation is a natural process in which DNA molecule is added to a methyl group so as to change its activity but not its sequence (Crimmins et al., 2024).

The first-generation of epigenetic clocks was developed by Steve Horvath and Gregory Hannum independently around the same time. Horvath demonstrated that biological age could be estimated by DNA methylation across multiple cell types, tissues, and species (Horvath, 2013) while Hannum developed a clock based on blood samples (Hannum et al., 2013). Integrating machine learning, both these clocks were able to predict biological aging or, even more accurately, epigenetic aging. This was compared to chronological aging, which divided people into accelerated agers vs. decelerated agers. Higher biological age as compared to chronological age were accelerated agers, and correspondingly, lower biological age compared to chronological age were decelerated agers (Crimmins et al., 2024).

While first-generation clocks predicted chronological age, it was with the advent of second-generation epigenetic clocks that incorporated clinical and lifestyle factors to determine health outcomes. GrimAge clock, a second-generation epigenetic clock, used an algorithm that incorporated plasma proteins and clinical factors such as chronological age, sex, and smoking pack-years in its calculations in predicting age-related clinical phenotypes and all-cause mortality. It has the strongest correlation between smoking and accelerated biological aging among the epigenetic clocks (Lu et al., 2019; McCrory et al., 2021).

Third and fourth generation epigenetic clocks have also been developed recently and are increasingly used in research. DunedinPoAm38 and DunedinPACE are the third-generation clocks that have been developed. In brief, they study the rate of change of epigenetic age rather than epigenetic age directly. Fourth-generation clocks, which have been introduced, are causal clocks that are composed of only those CpG sites that are believed to be causal in overall aging. They integrate the concept of Mendelian randomization to determine the causal relationship between CpG sites and outcomes (Crimmins et al., 2024).

Telomere length is another means to measure biological aging. Telomeres are the protective caps on the ends of the chromosomes. Their shortening occurs with progressive cell division or damage, and this attrition can lead to cellular senescence and, thus, a marker for biological aging. Though results have been inconsistent, it is useful when used in conjunction with other clocks. Other clocks being investigated are proteomic clocks (that measure plasma proteins), metabolomic clocks (that measure metabolites), transcriptomic clocks (that measure mRNA levels), and microbiomics clocks (that measure the microbiome in the gut) (Min et al., 2024).

## 4 Effect of tobacco on biological aging

Tobacco is among the most potent environmental accelerators of biological aging, exerting its effects through oxidative stress, chronic inflammation, and direct epigenetic modification. Cigarette smoke induces widespread alterations in DNA methylation, particularly hypomethylation at Aryl Hydrocarbon Receptor Repressor (AHRR) (cg05575921), F2RL3, and other CpG sites involved in xenobiotic metabolism and immune regulation. These methylation signatures not only serve as biomarkers of exposure but also mediate downstream biological effects that accelerate aging pathways, including endothelial dysfunction, mitochondrial injury, and genomic instability (Breitling et al., 2011; Zeilinger et al., 2013; Joehanes et al., 2016).

Epigenetic clocks—particularly GrimAge, which incorporates methylation-based surrogates for smoking pack-years and mortality-associated plasma proteins—consistently demonstrate epigenetic age acceleration of approximately 2–6 years in current smokers compared to non-smokers. This acceleration reflects the cumulative biological burden of smoking rather than mere exposure, with heavier and longer-term use producing proportionally greater effects (Lu et al., 2019; Joehanes et al., 2016; Cardenas et al., 2022).

Smoking also contributes to telomere shortening, further compounding cellular senescence and age-related morbidity (Astuti et al., 2017). Importantly, some methylation changes, such as those at AHRR, exhibit partial reversibility after smoking cessation, suggesting that biological aging induced by tobacco may be modifiable through behavioral and environmental interventions (Philibert et al., 2016). Thus, tobacco’s influence extends beyond its role as a carcinogen or cardiovascular risk factor—it acts as a systemic pro-aging agent, reshaping the epigenome and advancing the molecular clock of aging.

## 5 Counteracting and counterbalancing factors through the andorran perspective

**FIGURE 1:**
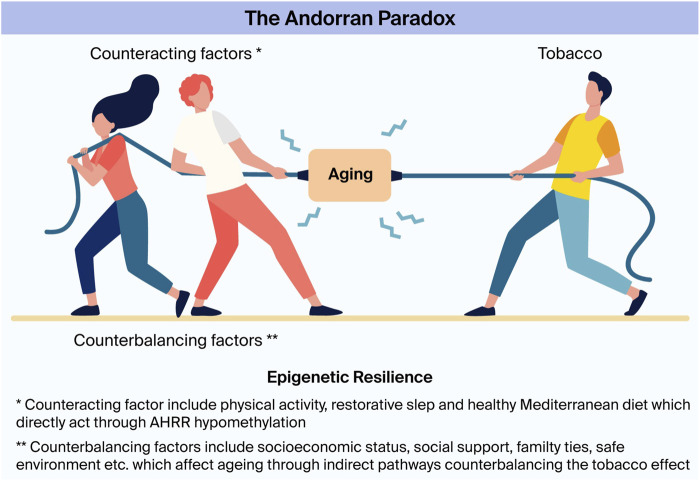
Schematic representation of the Andorran paradox, illustrating how protective lifestyle and environmental factors may counterbalance tobacco-associated biological aging and promote epigenetic resilience.

Tobacco smoking accelerates biological aging primarily through hypomethylation of AHRR, disrupting aryl hydrocarbon receptor signaling and promoting inflammation, immune dysregulation, atherosclerosis, and carcinogenesis (Bojesen et al., 2017; Langsted et al., 2020; Grieshober et al., 2020; Skov-Jeppesen et al., 2024). However, the biological consequences of smoking are not determined solely by exposure intensity. Instead, they reflect the dynamic balance between harmful influences and protective factors acting across molecular, physiological, and population levels. Within this framework, it is useful to distinguish between counteracting and counterbalancing factors, which together shape epigenetic resilience (Figure 1).

### 5.1 Counteracting factors: epigenetic and molecular modifiers of smoking-induced aging

Counteracting factors are exposures that directly modify smoking-associated molecular and epigenetic alterations, thereby influencing biological aging at the cellular level. These factors operate through epigenetic pathways—including DNA methylation, chromatin remodeling, inflammatory signaling, and mitochondrial function—and may partially attenuate or reverse smoking-induced epigenetic damage.

Mounting evidence demonstrates that lifestyle interventions can counteract tobacco-related epigenetic aging. Regular physical activity, restorative sleep, and adherence to Mediterranean dietary patterns have been associated with increased AHRR methylation and slower epigenetic clock progression (Pośpiech et al., 2024; Fernández-Carrión et al., 2023; Tsuboi et al., 2022). Importantly, diet and caloric balance are now recognized as central determinants of epigenetic aging, exerting effects comparable in magnitude to physical activity. Nutritional status influences conserved nutrient-sensing pathways, including sirtuins, AMP-activated protein kinase (AMPK), and insulin/insulin-like growth factor-1 (IGF-1) signaling, which are established hallmarks of aging biology (López-Otín et al., 2023).

Among these pathways, sirtuins—particularly SIRT1 and SIRT6—provide a plausible mechanistic bridge linking diet to epigenetic resilience. These nicotinamide adenine dinucleotide (NAD^+^)-dependent deacetylases respond to caloric moderation, fasting states, and dietary polyphenols, and regulate DNA repair, chromatin stability, inflammatory signaling, and mitochondrial function. Dysregulation of nutrient sensing accelerates epigenetic aging, whereas favorable metabolic states are associated with decelerated epigenetic clock progression and preserved genomic integrity (López-Otín et al., 2023).

In this context, Andorra’s Catalan–Mediterranean dietary pattern may play a counteracting role. Diets rich in fruits, vegetables, legumes, olive oil, and polyphenol-containing foods have been associated with favorable DNA methylation profiles and reduced epigenetic age acceleration, including modulation of smoking-sensitive loci such as AHRR (Tsuboi et al., 2022). This concept parallels observations from the historically described “French paradox,” wherein unexpectedly low cardiovascular mortality occurred despite relatively high intake of saturated fats (Renaud and de Lorgeril, 1992). Moderate red wine consumption—rich in polyphenols such as resveratrol—was proposed as a contributing factor, with experimental data demonstrating resveratrol-mediated activation of SIRT1 and downstream effects on epigenetic and metabolic pathways relevant to aging (Baur et al., 2006; Imai and Guarente, 2014). Although precise intake data are unavailable, acknowledging similar dietary patterns in Andorra strengthens the biological plausibility of diet-driven epigenetic counteraction.

Beyond adult lifestyle, epigenetic counteraction may also be shaped by early-life and transgenerational influences. Experimental models, such as the agouti mouse, demonstrate that nutritional exposures during embryogenesis induce persistent DNA methylation changes with lifelong metabolic consequences (Dolinoy et al., 2006; Waterland and Jirtle, 2003). Human studies—including the Dutch Hunger Winter and the Överkalix cohort—have shown that nutritional stress during fetal development and adolescence leaves durable epigenetic marks that influence cardiometabolic risk and, in some cases, extend across generations (Heijmans et al., 2008; Tobi et al., 2009; Pembrey et al., 2006). These findings suggest that populations do not enter adulthood from identical epigenetic baselines; instead, early-life nutritional environments may precondition resilience or vulnerability to later-life exposures such as tobacco.

While counteracting factors directly influence smoking-associated epigenetic alterations at the molecular level, they operate within a broader exposomic context. Even when molecular damage is not fully reversed, favorable social and environmental conditions may still mitigate the downstream health and longevity consequences of smoking, motivating the complementary concept of counterbalancing factors.

### 5.2 Counterbalancing factors: exposomic and population-level buffers of biological aging

Counterbalancing factors refer to social, environmental, and structural exposures that mitigate the population-level impact of smoking without necessarily reversing smoking-induced epigenetic changes at the molecular level. These factors influence biological aging indirectly by reducing cumulative stress burden, enhancing recovery capacity, improving disease prevention and management, and shifting the overall balance between harmful and protective exposures across the life course.

Large population studies underscore the importance of such counterbalancing influences. Analyses from the UK Biobank (∼500,000 participants) demonstrated that smoking, chronological age, and sex were the strongest accelerators of biological aging, whereas socioeconomic status, physical activity, and psychosocial wellbeing were consistently protective (Argentieri et al., 2025). Argentieri et al. identified 25 environmental and social exposures—including low socioeconomic status, physical inactivity, and psychological distress—as significant drivers of accelerated epigenetic aging (Argentieri et al., 2025). A meta-analysis by Oblak et al. confirmed smoking as the dominant contributor to GrimAge acceleration, while higher socioeconomic status and physical activity reliably decelerated epigenetic aging (Oblak et al., 2021). Hillmann et al. further demonstrated that greater perceived social support and more frequent contact with friends and family were associated with lower GrimAge and slower biological aging trajectories (Hillmann et al., 2023).

Andorra consistently ranks among the highest countries worldwide for Human development index (HDI) (Nations, 2025) and first in the Healthcare Access and Quality Index (The Lancet, 2025). Strong social cohesion, multigenerational family structures, low crime, and a low-stress environment characterize the Andorran way of living. High-quality healthcare access further might be reducing the downstream consequences of smoking by enabling early detection and effective management of tobacco-related disease, even in the presence of persistent exposure.

Taken together, counteracting and counterbalancing factors operate through distinct but complementary pathways. Counteracting mechanisms act at the molecular and epigenetic level to attenuate smoking-induced biological aging, whereas counterbalancing factors shape the broader exposome to reduce the clinical and population-level consequences of tobacco exposure. The Andorran paradox is therefore less a reflection of reduced biological harm from smoking and more an illustration of how a favorable exposomic environment—encompassing diet, caloric balance, nutrient-sensing biology, early-life conditioning, social structure, and healthcare access—can collectively buffer one of the most powerful accelerators of biological aging. This integrated perspective provides a coherent foundation for the concept of epigenetic resilience.

## 6 Epigenetic resilience: conceptual model and testable hypotheses

### 6.1 Conceptualizing epigenetic resilience

**FIGURE 2:**
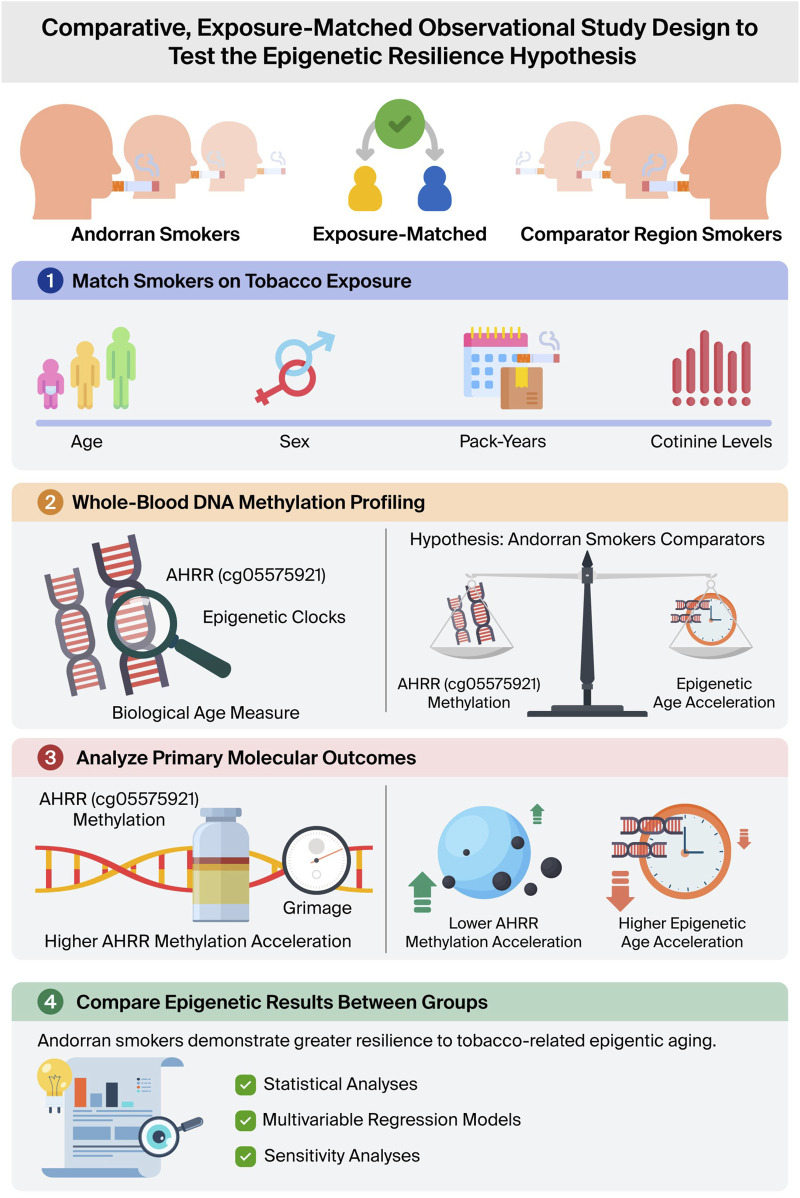
Exposure-matched observational study design evaluating epigenetic resilience in Andorran smokers through DNA methylation profiling and epigenetic aging biomarkers.

Epigenetic resilience refers to the capacity to maintain relatively stable biological aging trajectories, as measured by DNA methylation patterns and epigenetic aging clocks, despite sustained exposure to strong pro-aging influences such as tobacco. Rather than implying resistance to molecular damage, epigenetic resilience captures the extent to which biological aging remains constrained or decoupled from exposure burden at the population or individual level (Figure 2).

Importantly, epigenetic resilience is not a single exposure-dependent effect but a system-level property that emerges over time. It reflects differences in baseline epigenetic states, the ability of regulatory systems to accommodate chronic stress, and the persistence of aging trajectories across the life course. As a result, individuals and populations with similar risk exposures may exhibit markedly different patterns of biological aging.

This concept does not imply resistance to biological damage or preservation of normal physiology in the presence of harmful exposures. Instead, it reflects variability in the extent to which biological aging trajectories, as measured by epigenetic markers, are modulated by the interplay between adverse and protective factors.

### 6.2 The Andorran paradox as a natural experiment

The Andorran paradox provides a natural setting in which epigenetic resilience can be examined empirically. Despite one of the highest smoking prevalences worldwide, Andorra consistently demonstrates exceptional life expectancy and favorable health outcomes. This divergence between exposure burden and longevity suggests that biological aging trajectories in Andorra may be less accelerated than would be expected based on tobacco exposure alone.

Importantly, Andorra is particularly well-suited for studying epigenetic resilience because access to healthcare, socioeconomic conditions, and population genetic structure are relatively homogeneous compared with larger and more heterogeneous countries. This relative uniformity helps in reducing some sources of confounding when evaluating differences in biological aging in the presence of sustained exposure.

Together, these characteristics position Andorra as a natural experiment in which differences in biological aging can be examined independently of major structural inequities.

### 6.3 Integrated hypothesis and empirical strategy

#### 6.3.1 Hypothesis

Despite comparable tobacco exposure, smokers in Andorra might be exhibiting higher methylation at smoking-sensitive loci such as *AHRR* and lower epigenetic age acceleration than smokers residing in less favorable exposomic settings (Figure 2).

#### 6.3.2 Methodological approach to test the hypothesis

This hypothesis can be evaluated using a comparative, exposure-matched observational design. Adult current smokers could be recruited from Andorra and from one or more comparator regions characterized by a less favorable exposome. Participants could be matched or weighted on key determinants of tobacco exposure, including age, sex, smoking intensity (pack-years), and objective biomarkers such as cotinine, to ensure comparability of smoking burden across groups.

Whole-blood DNA methylation profiling could be performed using standardized platforms. The primary molecular outcomes would be (i) methylation at established smoking-associated CpG sites, with *AHRR* (cg05575921) as the principal locus of interest, and (ii) epigenetic age acceleration measured using smoking-sensitive epigenetic clocks, such as GrimAge. Group-level comparisons would then test whether smokers in Andorra demonstrate systematically higher *AHRR* methylation and lower epigenetic age acceleration than matched smokers from comparator populations.

Secondary analyses would assess within-Andorra variability, examining whether heterogeneity in epigenetic aging exists among smokers despite similar exposure levels. Multivariable regression models could be used to determine whether differences in epigenetic outcomes persist after adjustment for residual confounders, including socioeconomic indicators, comorbidities, and lifestyle factors. Sensitivity analyses excluding former smokers or stratifying by smoking duration would further test robustness.

Support for the hypothesis would be demonstrated by a consistent shift toward less accelerated epigenetic aging among Andorran smokers relative to matched external controls. Failure to observe such differences after rigorous exposure matching would argue against the presence of a measurable epigenetic resilience effect at the population level.

While Andorra is used here as an illustrative example based on the observed paradox, this framework is not specific to a single population and may be applied to any setting in which a high burden of adverse exposures coexists with unexpectedly favorable aging or health outcomes.

## 7 Limitations and scope of inference of the andorran paradox

The Andorran paradox described in this perspective is based on population-level observations and should be interpreted within the framework of ecological inference. While Andorra exhibits one of the highest life expectancies globally alongside a high prevalence of cigarette smoking, this observation does not imply equivalence in lifespan, biological aging, or disease risk between smokers and non-smokers within the same population. Nor does it suggest that the harmful biological effects of tobacco are neutralized at the individual level by favorable lifestyle or social factors. Rather, tobacco remains an important contributor to mortality in Andorra, underscoring that smoking-related harm persists at the individual level despite favorable population-level health indicators (Andorra, 2025).

At present, detailed stratified data comparing longevity, epigenetic clock acceleration, or smoking-associated DNA methylation markers between smokers and non-smokers in Andorra are limited. Similarly, there are insufficient data examining heterogeneity within the Andorran smoking population, such as differences in biological aging trajectories across gradients of physical activity, dietary adherence, socioeconomic status, healthcare utilization, or psychosocial support. These gaps limit the ability to draw causal or individual-level conclusions and raise the possibility of ecological fallacy if population-level patterns are overinterpreted.

Residual confounding from unmeasured factors—including survivor bias, mortality selection, health-seeking behavior, and heterogeneity in individual-level lifestyle adherence—cannot be excluded and may influence the observed population-level patterns.

Additionally, national life expectancy reflects aggregate survival across the entire population and may be influenced by demographic structure, migration patterns, healthcare access, and mortality from causes unrelated to smoking. These factors complicate direct attribution of population-level longevity to any single exposure or protective influence. As such, the present framework does not attempt to quantify the relative contribution of tobacco *versus* protective exposures to lifespan or healthspan.

Accordingly, the concept of epigenetic resilience proposed here should be viewed as a hypothesis-generating, population-level model that integrates existing epigenetic and exposomic literature. Its purpose is to motivate future studies capable of disentangling individual- and population-level effects using molecular aging markers, longitudinal designs, and cross-population comparisons, rather than to establish definitive causal relationships.

## 8 Conclusions and future directions

The Andorran paradox illustrates how protective lifestyle and social factors might potentially be buffering the harmful effects of smoking on biological aging. Despite one of the highest smoking prevalences worldwide, Andorra consistently ranks among the longest-lived populations. Mechanistically, tobacco accelerates aging through AHRR hypomethylation and epigenetic clock acceleration, yet Andorra’s exposome—including Mediterranean diet, physical activity, restorative sleep, high socioeconomic status, robust healthcare, and strong social cohesion—appears to counteract and counterbalance these effects.

Andorra may thus represent an illustrative example of resilience, highlighting the possibility that longevity reflects a balance between harmful exposures and protective influences. These observations are hypothesis-generating and warrant validation through individual-level molecular and longitudinal studies.

The broader implication is clear: if protective exposures can mitigate the biological impact of one of the strongest aging accelerators—tobacco—they may also buffer other risks such as poor diet, pollution, or psychosocial stress. Identifying and amplifying these decelerating factors can inform both public health policy and individual strategies to promote healthy aging.

## Data Availability

The original contributions presented in the study are included in the article/supplementary material, further inquiries can be directed to the corresponding author.
